# High‐Entropy Catalysis Accelerating Stepwise Sulfur Redox Reactions for Lithium–Sulfur Batteries

**DOI:** 10.1002/advs.202402497

**Published:** 2024-06-17

**Authors:** Yunhan Xu, Wenchuang Yuan, Chuannan Geng, Zhonghao Hu, Qiang Li, Yufei Zhao, Xu Zhang, Zhen Zhou, Chunpeng Yang, Quan‐Hong Yang

**Affiliations:** ^1^ Nanoyang Group Tianjin Key Laboratory of Advanced Carbon and Electrochemical Energy Storage School of Chemical Engineering and Technology Tianjin University Tianjin 300072 China; ^2^ Interdisciplinary Research Center for Sustainable Energy Science and Engineering School of Chemical Engineering Zhengzhou University Zhengzhou 450001 China

**Keywords:** electrocatalysis, high‐entropy alloys, lithium‐sulfur batteries, shuttle effect

## Abstract

Catalysis is crucial to improve redox kinetics in lithium–sulfur (Li–S) batteries. However, conventional catalysts that consist of a single metal element are incapable of accelerating stepwise sulfur redox reactions which involve 16‐electron transfer and multiple Li_2_S_n_ (n = 2–8) intermediate species. To enable fast kinetics of Li–S batteries, it is proposed to use high‐entropy alloy (HEA) nanocatalysts, which are demonstrated effective to adsorb lithium polysulfides and accelerate their redox kinetics. The incorporation of multiple elements (Co, Ni, Fe, Pd, and V) within HEAs greatly enhances the catalytically active sites, which not only improves the rate capability, but also elevates the cycling stability of the assembled batteries. Consequently, HEA‐catalyzed Li–S batteries achieve a high capacity up to 1364 mAh g^−1^ at 0.1 C and experience only a slight capacity fading rate of 0.054% per cycle over 1000 cycles at 2 C, while the assembled pouch cell achieves a high specific capacity of 1192 mAh g^−1^. The superior performance of Li–S batteries demonstrates the effectiveness of the HEA catalysts with maximized synergistic effect for accelerating S conversion reactions, which opens a way to catalytically improving stepwise electrochemical conversion reactions.

## Graphical abstract

High‐entropy alloy (HEA) nanocatalysts with multi‐metallic configurations are introduced to boost the multi‐step, multi‐intermediate S redox reactions in Li–S batteries. The HEAs greatly enhance the catalytic active sites, not only improving the adsorption capability for intermediate polysulfides but also accelerating the redox kinetics of sulfur species, thus enabling practical Li–S batteries with high capacity and low decay rate of 0.054% per cycle over 1000 cycles.

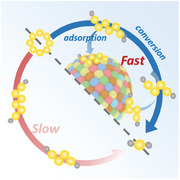

## 1 Introduction

Lithium–sulfur (Li–S) batteries have emerged as a promising alternative to lithium‐ion batteries in the field of electrochemistry, owing to their notable advantages such as high theoretical specific capacity (1675 mAh g^−1^), high energy density (2600 Wh kg^−1^), and cost‐effectiveness. However, the practical use of Li–S batteries faces several challenges including the dissolution of the lithium polysulfides (LiPSs) into the electrolyte and the shuttle effect of LiPSs.^[^ ^1^, ^2^ ^]^ The primary cause of these issues is the complex reaction mechanism of Li–S batteries, which involves multiple steps, multiple electrons, and multiple intermediate polysulfide products (Li_2_S_n_, n = 2–8).^[^ ^3^ ^]^ Therefore, intervening in the behavior of polysulfides to enhance the capacity and stability of Li–S batteries is of paramount importance. Additionally, it is indispensable to use lean electrolytes, instead of the excessive use of liquid electrolytes in most Li–S batteries, for high energy density, which, however, further challenges the S redox kinetics.^[^ ^2^, ^4^ ^]^ For addressing these challenges, various carbon materials have been explored physically adsorb polysulfides.^[^ ^5^ ^]^ However, their adsorption capacity remains insufficient for effectively inhibiting the shuttle effect and retaining capacity, especially in lean‐electrolyte batteries.^[^ ^6^ ^]^

Catalysis has been realized as a promising approach for Li–S batteries, with the dual objectives of adsorbing LiPSs and improving the kinetics of sulfur redox reactions.^[^ ^7^ ^]^ There are numerous kinds of catalysts applied in Li–S batteries, including metal‐based compounds ^[^ ^8^ ^]^, single atoms ^[^ ^9^ ^],^ and heterostructures.^[^ ^10^ ^]^ Most catalysts introduce one or two metal elements, such as Co, Fe, to accelerate the redox kinetics of polysulfides.^[^ ^11^ ^]^ However, due to the low number of active sites, the catalytic activity of these simple‐component catalysts is insufficient for the complex S redox reactions involving 16 electron conversion.^[^ ^12^ ^]^ In addition, the electrochemical stability of some catalysts is poor, which reduces the cycling stability of Li–S batteries.^[^ ^13^ ^]^ Consequently, in order to achieve catalysts with high catalytic activity, high stability, and applicability, multi‐component synergy has emerged as a prevalent design strategy in recent years, including multi‐phase synergy,^[^ ^14^ ^]^ multi‐metal synergy,^[^ ^15^, ^16^, ^17^ ^]^ and so on. High‐entropy alloys (HEAs), with five or more elements, are gaining increasingly high research interest as catalysts.^[^ ^18^, ^19^ ^]^ This is due to their rich combinatorial interface chemistry and homogeneous solid‐solution structure, which leverage the advantages of multiple elements to maximize multi‐metal synergy, resulting in high catalytic properties.^[^ ^20^ ^]^ The unique combinatorial chemistry properties of HEAs make them especially suitable for Li–S battery catalysis as they can be tailored according to the catalytic preference for multiphase and multi‐electron reactions of S redox.^[^ ^21^, ^22^ ^]^ In addition, HEAs have excellent thermodynamic stability due to their different atomic sizes, high configuration entropy, and sluggish diffusion properties,^[^ ^23^, ^24^, ^25^ ^]^ favoring the stability of long‐term cycling in Li–S batteries.

**Figure 1:**
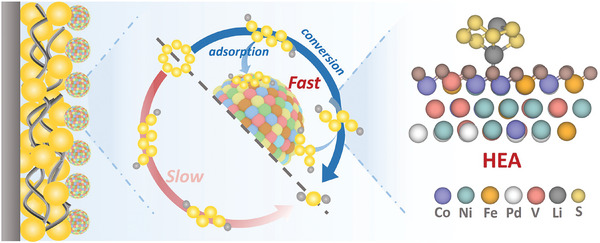
Schematics of stepwise conversion reactions of S in Li–S batteries with or without HEA nanocatalysts, which adsorb and catalyze LiPSs. The simulation optimized configuration of Li_2_S_6_ captured on the HEAs surface is shown as an example to illustrate the effect of the HEA nanocatalysts capturing LiPSs and suppressing shuttle effect.

Herein, we employ HEA nanocatalysts to accelerate stepwise sulfur redox reactions for Li–S batteries. We incorporated vanadium (V) and palladium (Pd) elements into the conventional transition metal alloy CoNiFe, thereby developing CoNiFePdV high‐entropy alloys by ultrafast Joule heating synthesis on carbon nanofibers (CNFs). The incorporation of V and Pd has demonstrated noticeable synergistic effects on the structural properties and electrochemical activity of HEAs. Specifically, V in the HEA catalyst significantly boosts active sites, and due to its synergistic interaction with Pd, there is a notable improvement in the LiPSs adsorption and catalysis ability, thereafter reducing the loss of the active sulfur material. In situ Raman analysis together with density functional theory (DFT) calculations demonstrates the interaction between HEAs and LiPSs, which effectively suppresses the dissolution of LiPSs, consequently inhibiting the shuttle effect (**Figure** **1**). As a result, the HEA‐based batteries exhibit a remarkable specific capacity of up to 1364 mAh g^−1^ at a rate of 0.1 C, along with excellent cycling stability and rate capability. The incorporation of CoNiFePdV high‐entropy alloys in the modification of Li–S batteries validates the feasibility of high‐entropy catalysis and presents design paradigms for high‐entropy catalysts, thereby highlighting the potential in advancing high‐performance batteries.

## 2 Results and Discussion

**Figure 2:**
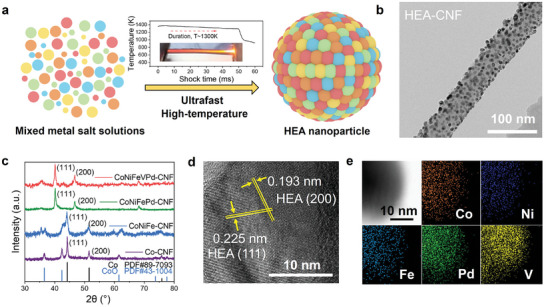
Formation and structural characterization of CoNiFePdV high‐entropy alloys. a) Schematic representation of HEAs; b) TEM image of the HEA nanoparticles loaded on CNFs; c) XRD patterns of HEAs, CoNiFePd, CoNiFe, and Co; d) High‐resolution TEM image of a HEA nanoparticle; e) STEM image and corresponding EDS mappings of Co, Ni, Fe, Pd, and V elements, respectively.

In this study, we use ultrafast Joule heating to fabricate HEAs. Compared with microwave heating,^[^ ^26^ ^]^ ball milling,^[^ ^27^ ^]^ and laser metal deposition,^[^ ^28^ ^]^ this method is effective because it quickly heats and cools the materials, which is necessary to form HEAs with their unique single‐phase structure. Moreover, the millisecond‐level short heating time also enhances the potential for synthesizing nanoscale metal particles with high specific surface area and multiple active sites.^[^ ^24^ ^]^ Dropping the mixed metal salts (Co^2+^, Ni^2+^, Fe^3+^, Pd^2+^, and V^3+^) with ethanol‐based solutions quantitatively onto the surface of the CNFs used as the loading substrate (Figure S1, Supporting Information). The experiment established a heating time of 50 ms (Figure S2, Supporting Information). Rapid and high‐temperature synthesis allows free metal ions to redistribute and alloy a uniform solid solution phase, characterized by a disordered chemical structure (**Figure** **2**a; Figure S3, Supporting Information). Additionally, we employed inductively coupled plasma (ICP) experiments to investigate the atomic ratios of these five metals in HEAs (Table S1, Supporting Information), and the atomic ratio of CoNiFePdV in the HEA is ≈1.9: 1.8: 1.8: 1.1: 0.9.

X‐ray diffraction (XRD) and transmission electron microscopy (TEM) results demonstrate the crystal face‐centered cubic (fcc) structure and nano‐sized particle morphology of HEAs. After ultrafast joule heating, nanoscale HEA particles with ≈10–15 nm in size were successfully dispersed on CNFs with defect concentrations (Figure 2b). These defects such as vacancies or structural irregularities present on the surface of CNFs can serve as nucleation points for the formation of dispersed particles, promoting the uniform distribution of HEAs.^[^ ^29^ ^]^ In addition, the interactions between the HEA particles and the defect sites on the CNFs affect the overall chemical and physical properties of the composites, which could potentially increase the catalysis activity and improve the structural stability. The XRD results of Co‐CNF exhibit two typical peaks at 44.2 and 51.5°, corresponding to the (111) and (200) planes of metallic cobalt with fcc structure (PDF#89‐7093). The peak of the (111) plane in CoNiFe shifts to a lower angle due to the embedment of Fe and Ni atoms in the metal Co lattice.^[^ ^30^ ^]^ For the CoNiFeV sample, CoNiFePd sample, and the HEA sample (Figure 2c; Figure S4, Supporting Information), the peak of the (111) plane has an obvious downshift without any new characteristic peaks appearing, which means that the doping of Pd increases the lattice constant of the metal, and the fcc structure remains without the phase separation.^[^ ^31^ ^]^ In addition, minor metal oxide peaks were detected by the XRD patterns (Figure 2c) and Raman spectra (Figure S5, Supporting Information).^[^ ^24^, ^32^ ^]^ The high‐resolution TEM and STEM images in Figure 2d,e further demonstrate that five metals (Co, Ni, Fe, Pd, and V) are uniformly distributed to form a single solid solution phase. The presence of well‐defined lattice fringes in the TEM image, with d‐spacing values of 0.225 and 0.193 nm corresponds to the (111) and (200) planes of HEAs, respectively. These d‐spacing values are larger compared to those of pure Co (0.205 and 0.177 nm), indicating lattice expansion in the HEAs due to the incorporation of multiple elements, corresponding with the downshift of the peak position in XRD patterns (Figure 2c).^[^ ^33^ ^]^ Compared with CoNiFeV, CoNiFe, and Co nanoparticles, the doping of Pd significantly increases the interatomic spacing of the alloy, because of the highest electronegativity (2.20) and atomic radius (137 pm).^[^ ^34^ ^]^ The increase in lattice spacing results in the lattice distortion of the alloys.^[^ ^35^ ^]^ The uniform distribution of metal elements in the HEA phase (Figure 4e) suggests a high degree of atomic mixing. All the above results demonstrate that the single solid solution structure of HEAs was successfully synthesized with a highly disordered configuration, which enhances the structural stability and can lead to unique properties such as enhanced catalytic activity and structural stability of the HEA.

**Figure 3:**
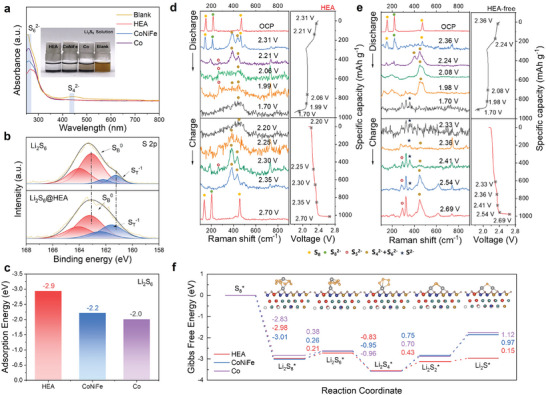
Catalytic performances of HEAs and computational studies of polysulfide conversions. a) Photograph of adsorption tests of Li_2_S_6_ with different catalysts and corresponding UV‐vis spectra; b) S 2p XPS of Li_2_S_6_ before and after adsorption by HEAs; c) Adsorption energies of Li_2_S_6_ on HEA, CoNiFe and Co surfaces; d) In situ Raman spectra of the HEA‐based S cathode at different voltage states and the corresponding charge‐discharge profiles. e) In‐situ Raman spectra of HEA‐free S cathodes at different voltage states and the corresponding charge‐discharge profiles; f) Energy profiles for the reduction of sulfur species on HEAs, CoNiFe, and Co.

To investigate the adsorption of HEAs on LiPSs, we conducted adsorption tests by adding an equal amount of HEAs, CoNiFe, and Co materials into a Li_2_S_6_ solution and aging for 24 h. Upon being adsorbed by the HEAs, the Li_2_S_6_ solution becomes colorless and transparent, whereas the color of Li_2_S_6_ adsorbed by CoNiFe and Co metals only became lighter in color. UV‐visible spectroscopy (**Figure** **3**a) further confirms this empirical observation, clearly demonstrating the superior adsorption capability of HEAs for LiPSs. The nanoscale nature of HEA materials confers upon them a heightened specific surface area and a profusion of adsorption sites on their surfaces, thereby facilitating the efficient adsorption of LiPSs molecules. Additionally, we investigated the chemical interactions between LiPSs and HEAs by X‐ray photoelectron spectroscopy analysis (XPS). The XPS spectrum of Li_2_S_6_ exhibits two distinct peaks of S 2p_3/2_, corresponding to the terminal sulfur (S_T_^−1^) and bridging sulfur (S_B_^0^) species, with binding energies of 161.28 and 163.08 eV, respectively (Figure 3b).^[^ ^36^, ^37^ ^]^ After the adsorption of HEAs, both peaks shifted toward higher binding energy. The shift of the Co 2p, Ni 2p, and V 2p peaks to lower binding energy after adsorption suggests the transfer of electrons from polysulfides to the metal active sites provided by Co, Ni, and V during the adsorption of LiPSs on HEAs (Figure S6, Supporting Information).

Furthermore, we used Li_2_S_6_ as a representative LiPSs for DFT calculations to compare the respective adsorption energies on HEA‐(111), CoNiFe‐(111), and Co‐(111) surfaces (Figure 3c). To start with a stable HEA structure, we first employed Monte Carlo (MC) simulations to optimize the positions of metal atoms in the HEA and CoNiFe models (Figure S7, Supporting Information).^[^ ^38^ ^]^ As the precursors of HEAs (metal nitrates) contain oxygen and may leave residual oxygen on the surface. Subsequently, we surface‐functionalize the pure metal substrate to obtain an oxygen‐terminated surface. Figure S8 (Supporting Information) demonstrates that the adsorption of oxygen atoms and O_2_ is spontaneous on three types of metal substrates likewise, *O_2_ → *2O (* represents the metal substrate) with a sufficient negative ∆G [(−4.86) − (−2.83 eV)], indicating that surface passivation with the addition of oxygen on the substrate is entirely spontaneous and reasonable. According to DFT calculations, the adsorption energy of Li_2_S_6_ on HEA is −2.9 eV, much more negative than those on CoNiFe and Co (−2.2 and −2.0 eV, respectively), indicating that the integration of V and Pd has beneficially enhanced the catalyst's affinity for LiPSs, which is consistent with the XPS results. The above computations demonstrate the existence of a chemical bondage between polysulfides and HEAs. This electron transfer process benefits from the high specific surface area and abundant adsorption sites of HEAs, which enhance the adsorption capacity of polysulfides.

In order to gain further insights into the conversion process of LiPSs and investigate the inhibitory effect of HEAs on the polysulfide shuttle behavior, we monitored the charge‐discharge process of Li–S batteries with or without HEA catalysts by in situ Raman spectroscopy with a 532 nm laser. During the discharge process, Raman signals corresponding to S_8_ (150.8 and 471.9 cm^−1^) and S_8_2^−^ (215.5 cm^−1^) ^[^ ^39^ ^]^ are detected at the open circuit potential (OCP). During discharge, the intensity of S_8_ and S_8_2^−^ peaks diminish, and the S_4_2^−^ + S_6_2^−^ signal at 391.5, 443.1 cm^−1^ and the S_5_2^−^ signal at 269.9 cm^−1^ gradually increase. These polysulfide peaks are still detected during the charging process, and at the end of the charge (2.70 V), the prominent peaks of S_8_ and S_8_2^−^ reappear (Figure 3d,e). In contrast, in the S cathode without HEA nanocatalysts, Raman results indicate the presence of a Li_2_S peak at 357.1 cm^−1^ during the charge–discharge process, indicating loss of active sulfur materials and degradation of battery performance.^[^ ^40^ ^]^ Furthermore, at the end of the charging process, the signals corresponding to S_8_ and S_8_2^−^ are not detected, suggesting a severe polysulfide dissolution effect and decreased utilization of active sulfur materials. The interaction between HEA nanocatalysts and LiPSs can account for the observed changes in the Raman spectra. HEAs accelerate the conversion of polysulfides to lower‐order species during discharge, reducing the formation and accumulation of higher‐order polysulfides that contribute to the shuttle effect. The re‐appearance of S_8_ and S_8_2^−^ peaks at the end of the charge process suggests the reversible conversion of lower‐order sulfur species back to higher‐order polysulfides. We further conducted XPS characterizations for sulfur species on the surface of the cycled electrodes (Figure S9, Supporting Information), confirming the accelerating effect of HEAs on the S redox kinetics, which is consistent with the in situ Raman results.

We utilize the Gibbs free energies of each reaction step to investigate the catalytic conversion process of S_8_ into Li_2_S by HEAs during discharge (Figure 3f; Figure S10, Supporting Information). The intermediate reduction steps from S_8_ to Li_2_S show similar trends in ΔG evolution. The first step from S_8_ to Li_2_S_8_ and the third step from Li_2_S_6_ to Li_2_S_4_ are both spontaneous. In the remaining three steps, the processes from Li_2_S_8_ to Li_2_S_6_, Li_2_S_4_ to Li_2_S_2_, and Li_2_S_2_ to Li_2_S are all endothermic. Among these reaction steps, the rate‐determining step depends on the last two steps. For HEA, the rate‐determining step is the second last step from Li_2_S_4_ to Li_2_S_2_, with a ΔG value of 0.43 eV; while for Co and CoNiFe, the rate‐determining step is the final step from Li_2_S_2_ to Li_2_S, with ΔG values of 1.12 eV and 0.97 eV, respectively. Thus, HEA exhibits the fastest kinetic process with the smallest ΔG value for the rate‐determining step, effectively reducing the reaction energy barrier required for sulfur redox reactions and promoting discharge reactions, thereby enhancing the electrochemical performance of Li–S batteries. This explains the above experimental results and highlights the effectiveness of the high‐entropy alloy catalysis for accelerating stepwise sulfur redox reactions. Considering both adsorption and catalysis ability, HEAs are the most promising catalysts for suppressing the shuttle effect in Li–S batteries, which can increase the utilization of active sulfur materials and improve the kinetics of S conversion reactions.

**Figure 4:**
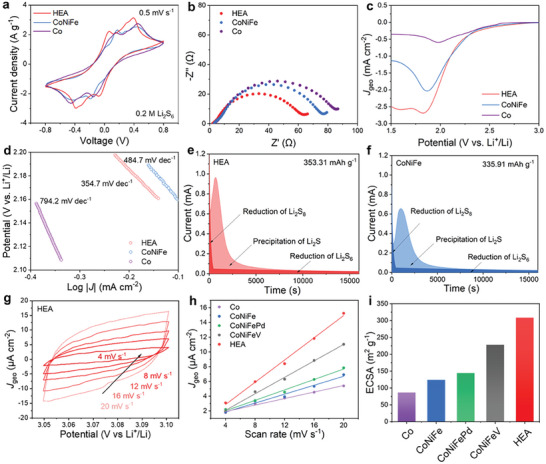
Electrochemical tests of catalytic performance. a) CV curves and b) EIS plots of different symmetrical cells; c) LSV profiles and d) corresponding Tafel plots of the sulfur reduction reaction. e,f) Li_2_S precipitation profiles of HEA and CoNiFe electrodes; g) CV curves of HEAs tested in the non‐faradic range; h) C_dl_ results of the various catalysts obtained from CV; i) ECSA values of the various catalysts.

We systematically investigated the catalytic activities of CoNiFePdV HEA, CoNiFeV, CoNiFePd, CoNiFe, and Co for the transformation of long‐chain polysulfides. Cyclic voltammetry (CV) was performed at a scan rate of 5 mV s^−1^ over a potential range of −0.8 to 0.8 V for symmetrical cells (**Figure** **4**a; Figure S11, Supporting Information). Each curves reveal two pairs of redox peaks, and compared with CoNiFe and Co‐CNT, the CV curve of HEA‐CNT‐based cell demonstrates a lower redox overpotential (0.06 V, from Li_2_S to Li_2_S_6_; and 0.39 V, from Li_2_S_6_ to S_8_) and a higher peak current density (2.23 and 3.15 A g^−1^), indicating the superior catalytic activity of HEAs. This enhanced catalytic activity accelerates the redox kinetic process between Li_2_S_6_ and Li_2_S. Electrochemical impedance spectroscopy (EIS) can confirm the improved catalysis activity of HEAs. In the high‐frequency region (Figure 4b; Figure S11, Supporting Information), the HEA‐CNT electrodes exhibit a smaller charge transfer resistance (*R*_ct_),^[^ ^41^ ^]^ indicating faster movement of charges at the interface between the electrode and the electrolyte, thanks to the catalytic effect of the HEAs.

We tested the role of HEA, CoNiFe, and Co catalysts by linear sweep voltammetry (LSV) and the corresponding Tafel plots in the S reduction process (Figure 4c,d). The Tafel slope provides valuable insights into the catalytic performance of the catalysts. A lower Tafel slope indicates a higher exchange current density and LiPSs conversion rate, indicating superior catalytic activity.^[^ ^42^ ^]^ HEA nanocatalysts exhibit the smallest slope value of 354.7 mV dec^−1^ compared with 484.7 and 794.2 mV dec^−1^ for CoNiFe and Co, respectively (Figure 4d). Considering the multi‐element composition of HEAs, we can attribute its excellent catalytic ability to the synergistic catalysis effects of five metals. Furthermore, in order to explore the catalytic ability of different catalysts for the reductive formation of Li_2_S, the potentiostatic discharge experiment was designed to research the precipitation of Li_2_S. Figure 4e,f and Figure S12 (Supporting Information) display that the HEA catalyst exhibits the largest capacity for Li_2_S precipitation (353.31 mAh g^−1^), which indicates that the HEA catalyst is highly effective in facilitating the conversion of S species to Li_2_S during the discharge process. Additionally, HEAs demonstrate the earliest precipitation time and the highest current. The early precipitation time suggests that the nucleation of Li_2_S occurs more readily on the surface of HEAs, indicating its favorable catalytic properties. The higher current indicates a faster growth rate of Li_2_S on the catalyst surface, further confirming its enhanced catalytic activity.

Through a detailed analysis of the results, we find that during the S redox reaction processes, V has a greater impact on the reaction kinetics than other elements. To analyze the difference in the effect of integration in V and Pd on the active sites and calculate the electrochemical active surface area (ECSA), we tested different catalyst film electrodes by CV from 4 to 20 mV s^−1^ (Figure 4g; Figure S13, Supporting Information). Figure 4h shows that double‐layer capacitance (C_dl_) for different catalysts was obtained by linearly fitting the cyclic voltammetry curves corresponding to the non‐Faraday interval double‐layer capacitance currents at different sweep speeds. According to Equation 1, C_s_ represents specific capacitance, C_dl_ positively correlates with the ECSA, and this parameter gives an indication of the catalytic activity center of the material. Usually, the higher value of C_dl_ indicates that the catalytic activity center is more abundant and the activity of the material is better.^[^ ^43^, ^44^ ^]^

(1) $$\mathrm{ECSA}\;=\frac{C_{\mathrm{dl}}}{C_{s}}\;$$

HEAs have the highest electrochemical active surface area (308.86 m^2^ g^−1^), followed by CoNiFeV (228.56 m^2^ g^−1^), CoNiFePd (144.60 m^2^ g^−1^), CoNiFe (124.12 m^2^ g^−1^), and Co (86.80 m^2^ g^−1^) (Figure 4i). Thus, we can see that the ability of V to increase catalysis active sites is much greater than that of Pd. Hence, these results illustrate that the CoNiFePdV high entropy alloy catalyst, whose catalysis activity is greatly enhanced, can strengthen the LiPSs conversion kinetics and increase the utilization efficiency of sulfur in Li–S battery.

**Figure 5:**
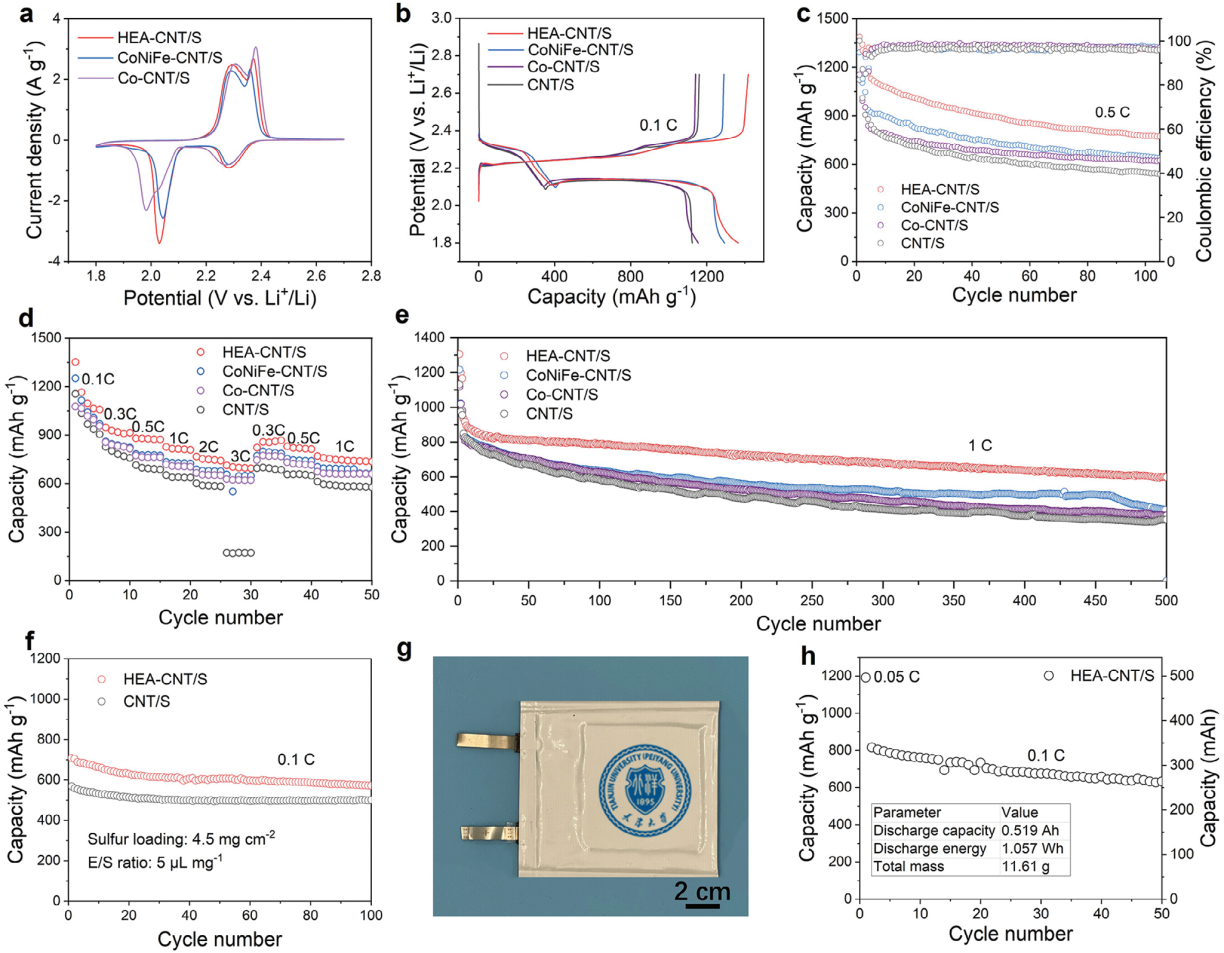
Electrochemical performance. a) CV curves of HEA‐CNT/S, CoNiFe‐CNT/S and Co‐CNT/S at 0.1 mV s^−1^; b) Charge–discharge curves of different S cathodes at 0.1 C; c) Cycling performance of different electrodes at 0.5 C; d) Corresponding rate performance at different specific current; e) Pro‐longed cycle life of different sulfur cathodes at 1 C; f) Cycling performance of HEA‐CNT/S and CNT/S (sulfur loading = 4.5 mg cm^−1^ and E/S = 5 µL mg^−1^); g) Optical photograph of Li–S pouch cell from the top view; h) Cycling performance at 0.1 C with the key battery performance parameters for Li–S pouch cell.

To examine the functionality of HEAs in Li–S batteries, we tested the electrochemical performance by pairing the S/CNT cathode, HEA‐based interlayer, and Li anode (Figure S14, Supporting Information). The CV curves collected at a scanning rate of 0.1 mV s^−1^ (**Figure** **5**a) indicate that all the cells exhibit two well‐defined cathodic peaks and two anodic peaks, corresponding to the multistep reduction process from S to Li_2_S_2_/Li_2_S and the reverse process.^[^ ^45^ ^]^ The HEA‐based cell shows significant cathodic peaks at 2.28 and 2.03 V, and anodic peaks at 2.30 and 2.37 V. The HEA‐based cell possesses a narrower polarization voltage and a higher peak intensity than CoNiFe‐based and Co‐based cells, suggesting its superior electrochemical kinetics. The EIS test results in Figure S15 (Supporting Information) also demonstrate that HEA‐CNT/S has a smaller charge transfer impedance. These findings indicate that the HEA‐based cells have improved electrochemical performance, suggesting their potential for enhancing the performance of Li–S batteries. Furthermore, the synergistic effects of V and Pd, as indicated by CV and EIS analyses with multiple control samples without V or Pd, lead to a significant enhancement in the electrochemical behavior of HEAs (Figure S16, Supporting Information).

The galvanostatic charge‐discharge curves of HEAs, CoNiFe, Co, and no catalyst electrodes (Figure 5b) were recorded at 0.1 C (1 C corresponds to 1675 mAh g^−1^). By comparison, the overpotential of HEA‐CNT/S cathodes (0.245 V) is significantly lower than that of CoNiFe‐CNT/S cathodes (0.258 V) and Co‐CNT/S cathodes (0.268 V), reflecting its rapid reaction kinetics of HEAs. Moreover, the HEA catalyst provides an initial specific capacity of 1164.7 mAh g^−1^ at 0.5 C and stability after 100 cycles, which is higher than that of CoNiFe catalysts and Co catalysts (Figure 5c) because of the incorporation of V and Pd. HEAs also enhance the rate capability. The HEA‐based cells achieve the specific discharge capacities of 1352, 948, 880, 826, 767, and 713 mAh g^−1^ at 0.1 C, 0.3 C, 0.5 C, 1 C, 2 C, and 3 C, respectively (Figure 5d). Notably, the capacity of HEA‐based cells recovers to 823 mAh g^−1^ as the current rate is shifted back to 0.3 C. HEAs also exhibit excellent stability in long‐term cycling. At a 1 C rate, Li–S batteries with HEA nanocatalysts can maintain a discharge specific capacity of 700 mAh g^−1^ after 500 cycles. At a 2 C rate, over 1000 cycles, the batteries experience a tiny capacity fading rate of 0.054% per cycle (Figure 5e; Figure S17, Supporting Information). These results highlight the superior electrochemical properties of HEA nanocatalysts and its potential for enhancing the performance of Li–S batteries.

To explore the practical potential, the electrochemical behaviors of HEA‐based cells under raised sulfur loading and limited electrolyte. This work tested two different E/S ratios of HEA‐based batteries, with a sulfur loading of 4.5 mg cm^−^^2^. The cells with HEA catalysts exhibit a higher specific capacity compared to those without catalysts under an E/S of 10 µL mg^−1^ (Figure S18, Supporting Information). To further demonstrate the superiority of the HEA catalyst, the cells under lean electrolyte (E/S = 5 µL mg^−1^) were evaluated (Figure 5f; Figure S19, Supporting Information). Encouragingly, the charge–discharge curve still shows an obvious double‐platform discharge profile, and these HEA‐based cells demonstrate excellent cycling performance, maintaining a specific capacity of 571.9 mAh g^−1^ even after 100 cycles. This result demonstrates that the catalytic ability of HEAs improves the electrochemical performance of Li–S batteries even under challenging operating conditions such as high sulfur loading or limited electrolyte availability.^[^ ^46^ ^]^ Furthermore, we fabricated a multi‐layer Li–S pouch cell with a large capacity (Figure 5g,h; Figure S20, Supporting Information), and assembled the multi‐layer pouch cell with a length and width of 1 cm and 3.5 cm. The typical discharge profiles are still achievable, with an initial specific capacity of up to 1192 mAh g^−1^ at 0.05 C. The specific energy of the pouch cell is calculated to be 297 Wh kg−1, which showcases its competitive potential in energy storage applications (Figure S21, Supporting Information). The excellent catalytic properties and stability performance of HEAs make them suitable for Li–S batteries, especially in lean‐electrolyte systems.

## 3 Conclusion

In summary, we successfully designed CoNiFePdV HEA nanocatalysts and applied them to the cathode of Li–S batteries. The doping of CoNiFePdV with five metals effectively accelerates the redox reactions of sulfur species involving multiple electrons and multiple steps in Li–S batteries. Additionally, the incorporation of V significantly increases the specific surface area of HEA nanocatalysts, thereby enhancing LiPSs adsorption ability. Benefiting from these synergistic merits, the modification of Li–S batteries with HEAs resulted in notable advancements in cell cyclability, and rate capability. HEA‐catalyzed Li–S batteries deliver a high capacity of 1364 mAh g^−1^ at 0.1 C and have very small capacity fading of 0.054% decay per cycle for 1000 cycles at 2 C, much better than that of the HEA‐free batteries. More encouragingly, the HEA‐modified Li–S battery with a low E/S ratio of 5 µL mg^−1^ still maintains a high specific capacity (571.9 mAh g^−1^) after 100 cycles. This study demonstrates the potential of HEAs in advancing high‐performance Li–S batteries and contributes to the development of sustainable and efficient energy storage technologies. Future studies on high‐entropy catalysis are expected to deepen the theoretical understanding of the synergistic effect of HEAs, reduce dependence on rare elements, and enhance structural and cycling stability.

## Conflict of Interest

The authors declare no conflict of interest.

## Author Contributions

Y.X. and W.Y. contributed equally to this work. Q.‐H.Y. proposed the project, C.Y., Y.X. conceived the idea, and C.Y., Q.‐H.Y. supervised the project. C.G., Y.Z., and Q.L. performed the characterizations and electrochemical measurements. W.Y., X.Z., and Z.Z. performed DFT calculations and data analysis. Y.X., C.G., and H.H. contributed to the structural and performance analysis. Y.X., C.Y., and Q.‐H.Y. organized and wrote the manuscript. All authors contributed to the discussion and revision of the manuscript at all stages.

## Supporting information

Supporting Information

## Data Availability

The data that support the findings of this study are available in the supplementary material of this article.
