# Supplementary material for: High‐Entropy Catalysis Accelerating Stepwise Sulfur Redox Reactions for Lithium–Sulfur Batteries
Source: Adv Sci (Weinh). 2024 Jun 17;11(31):2402497. doi: 10.1002/advs.202402497 (PMC11336958; doi:10.1002/advs.202402497)
Supplement: Supplementary file 1 — Supporting Information [file ADVS-11-2402497-s001.pdf]

ADVANCED SCIENCE
Open Access

Supporting Information

for *Adv. Sci.*, DOI 10.1002/advs.202402497

High-Entropy Catalysis Accelerating Stepwise Sulfur Redox Reactions for Lithium–Sulfur Batteries

Yunhan Xu, Wenchuang Yuan, Chuannan Geng, Zhonghao Hu, Qiang Li, Yufei Zhao, Xu Zhang, Zhen Zhou, Chunpeng Yang* and Quan-Hong Yang*

Supporting Information

High-Entropy Catalysis Accelerating Stepwise Sulfur Redox Reactions for Lithium-Sulfur Batteries

Yunhan Xu, Wenchuang Yuan, Chuannan Geng, Zhonghao Hu, Yufei Zhao, Qiang Li, Xu Zhang, Zhen Zhou, Chunpeng Yang*, Quan-Hong Yang*

Y. Xu, C. Geng, Z. Hu, Y. Zhao, Q. Li, Dr. C. Yang, Dr. Q.-H. Yang

Nanoyang Group

Tianjin Key Laboratory of Advanced Carbon and Electrochemical Energy Storage

School of Chemical Engineering and Technology

Tianjin University

Tianjin 300072, China

E-mail: cpyang@tju.edu.cn

W. Yuan, Dr. X. Zhang, Dr. Z. Zhou

Interdisciplinary Research Center for Sustainable Energy Science and Engineering

School of Chemical Engineering

Zhengzhou University

Zhengzhou 450001, China

Email: zzuhangxu@zzu.edu.cn

Experimental Procedures

Synthesis of CNFs: PAN was dissolved in DMF to a concentration of 10 wt%. Subsequently, electrospinning was conducted at a voltage of 15 kV, spinning distance of 15 cm. The electrospun fibers were collected on a rotating drum (at a speed of 80 rpm). The PAN nanofibers were stabilized in air at temperature of 523 K for two hours before being carbonized for two hours in Ar at temperature of 1073 K. Finally, the CNF was plasma activated.

Synthesis of HEAs, CoNiFeV, CoNiFePd, CoNiFe, and Co: Firstly, a predetermined amount of cobalt nitrate hexahydrate (29.1 mg), nickel nitrate hexahydrate (29.1 mg), iron (III) nitrate nonahydrate (40.4 mg), palladium chloride (17.7 mg) and vanadium chloride (15.7 mg) were dissolved in ethanol (1 mL) as solvent to prepare 0.1 mol L^{-1} precursor salt solutions respectively. Precursor solutions for each metal (Co, Ni, Fe, Pd, V) were individually measured at 100 μL, followed by thorough mixing through a 10-min ultrasonication process to achieve a well-blended precursor salt solution for HEAs formation. The plasma-activated CNFs were cut into rectangular shapes measuring 0.5×3.5 cm, and drop-coated with 100 μL of mixed salt solution onto each rectangle sample. The samples were then dried at 60 °C before being transferred to a Joule heating stage inside the glovebox. An electric voltage of 100 V with a current of 1 A was applied for a duration of 50 ms. Other samples were processed in the same manner as described above, except that the precursor salt solutions were different and were made by mixing equal amounts of different metal salt solutions selected as required.

Synthesis of S/CNT cathodes: Firstly, carbon nanotubes and sulfur powder were mixed evenly according to the mass ratio of 1:4, and then reacted at 155 °C for 12 h to prepare cathode materials. The cathode was obtained by grounding the S/CNT, CNT and PVDF (7:2:1, in mass ratio) in NMP solvent to form a mud mixture. And the mud was bladed on Al foil, dried at 55 °C for 12 h. The sulfur content of the ordinary

electrodes is 1 mg cm^{-2}. The high-sulfur-loaded cathode was obtained by grounding in CMC/SBR solvent, and the mud was bladed on Al foil, dried at 35 °C for 24 h followed by 55 °C for 12 h. The sulfur loading is 4.5 mg cm^{-2}. The prepared HEAs, CoNiFe, and Co catalysts were thoroughly ground in equal amounts with CNTs, followed by further grinding in NMP for 15 min to form a mud mixture. The mud was then dropped onto the pre-coated S/CNT cathode and bladed to create a 5~6 μm interlayer, followed by drying in an oven for 12 h. The loading of the modified interlayer was around 0.3 mg cm^{-2}.

Preparation of Li-S pouch cells: The sulfur cathodes used for pouch cells, S/CNTs (64 wt% sulfur content), conductive carbon (26 wt%, CNTs), LA133 binder (5 wt%) in solid content of 0.2 g mL^{-1} (H_2O) were well ground and subsequently dispersed. The obtained slurry was coated on both sides of a carbon-coated aluminum foil with a thickness of 800 μm each side, which was then transferred to a 35 °C heated plate to evaporate the H_2O for over 6 h. HEAs-CNF was mixed with an equivalent amount of CNTs, and the resulting mixture was thoroughly ground by dropwise addition of a measured quantity of NMP for 20 min to obtain a slurry. The slurry was then coated onto both sides of the positive electrode surface, followed by drying at 60 °C for at least 12 h. This sulfur electrode with the catalyst was used as the cathode, lithium foil (100 μm) was used as the anode, an alumina film was used as the separator, and Al-plastic film was used as packaging material. The electrolyte was DOL/DME (1:1, by volume) with 1 M LiTFSI and 5.0 wt% $LiNO_3$ additives. The assembly process was a multi-layer superposition, and both sides of the cell were lithium foil because of the double-coating cathode. The sulfur loading in the pouch cell is 434 mg, and the amount of electrolyte used is 2 mL.

Lithium polysulfide adsorption tests: 100 mM Li_2S_6 solution was firstly prepared by dissolving Li_2S and S at the mole ratio of 1:5 in a DOL/DME mixed solvent, and stirring at 60 °C for 24 h. Subsequently, the as-prepared Li_2S_6 solution was diluted to 2 mM by DME and 10 mg catalysts (HEAs, CoNiFe, Co) was

added to immerse for 12 h.

Physical characterization: The crystal structures of different catalysts were confirmed by XRD (Rigaku Smartlab). The micromorphologies were characterized using TEM (JEM–F200). XPS data were recorded by an ESCALAB 250Xi (Thermo Fisher) with a monochromatic Al Ka source to analyze the surface species and their chemical states. UV–vis adsorption tests were carried out by a Thermo UV-visible spectrophotometer. Coin cells with a quartz window and a hole in the stainless steel were used for in situ Raman spectroscopy analysis at 532 nm Laser. The S cathode was prepared by thoroughly mixing CNT-S powder (70 wt.%), CNT (10 wt.%), HEAs (10 wt.%), and PTFE (10 wt.%) followed by rolling it into sheets and pressing it onto foam nickel, then drying at room temperature for 48 hours.

Preparation of symmetric cells: Li_2S_6 electrolyte was prepared by the overnight reaction of Li_2S and S with a molar ratio of 1:5. Two identical CP electrodes were assembled, and 40.0 μL Li_2S_6 electrolyte was added. The CV and EIS test of symmetric cell was tested on the electrochemical workstation (PARSTAT MC), CV curves were obtained at a scan rate of 10 mV s^{-1} with a voltage range of -0.8 to 8 V.

Electrochemical characterization: LSV was tested by using a Bio-Logic workstation coupled with the RDE technique. The electrochemical test was performed in a three-electrode open-cell in the voltage range of 3.0 to 1.5 V. ECSA were tested by cyclic voltammetry from 4 mV s^{-1} to 20 mV s^{-1}. The CV was acquired on the PARSTAT MC workstation in the potentials of 1.7-2.8 V, and the EIS plots were collected within a frequency range of 0.01 Hz to 100 kHz. Neware battery test station was used to test the galvanostatic charge-discharge and rate performance. Li_2S nucleation tests use the CP working electrode with catalysts (HEAs, CoNiFe, Co). Routine electrolyte (20 μL) and Li_2S_8 electrolyte (20 μL) were added in the anode and cathode sides, respectively. The cells were galvanostatically discharged to 2.06 V, and then discharged potentiostatically at 2.02 V for 15000 s.

Computational details: Monte Carlo (MC) simulations were performed to build the initial structure of high-entropy alloys (HEAs), using the large-scale atomic/molecular massively parallel simulator (LAMMPS) package with embedded atom model (EAM) potential.[1] The distribution of atoms in HEAs was thus optimized by random selection and move of them, where the acceptance probability of each MC move was determined according to the Metropolis criterion.[2] After obtaining the optimized structure of HEAs from MC, density functional theory (DFT) computations were performed within using the Vienna *ab initio* simulation Package (VASP) to predict the catalytic performance of the material.[3] The Perdew-Burke-Ernzerhof (PBE) functional within the generalized gradient approximation (GGA) was used to describe the exchange-correlation interaction.[4] The cutoff energy of the plane wave basis set was set to 450 eV. A vacuum space of 15 Å was applied along the z-direction to avoid periodic interactions. DFT-D3 functional was adopted for the van der Waals (vdW) interactions.[5]

The binding energy was evaluated by the following expression:

$$E_b = E_{system} - E_{HEA} - E_{LixSy}$$

where E_{system} is the energy of the adsorbed system, and EHEA and E_{LixSy} are the energies of individual HEA catalytic substrate and polysulfides, respectively.

The Gibbs free energy (ΔG) for each step was calculated as below:

$$\Delta G = \Delta E + \Delta ZPE - T\Delta S$$

where ΔE, ΔZPE, and TΔS are the energy difference based on DFT calculations, zero-point energy (ZPE), and entropy change between the products and reactants, respectively. The ZPE and entropic corrections were calculated with VASPKIT.[6]

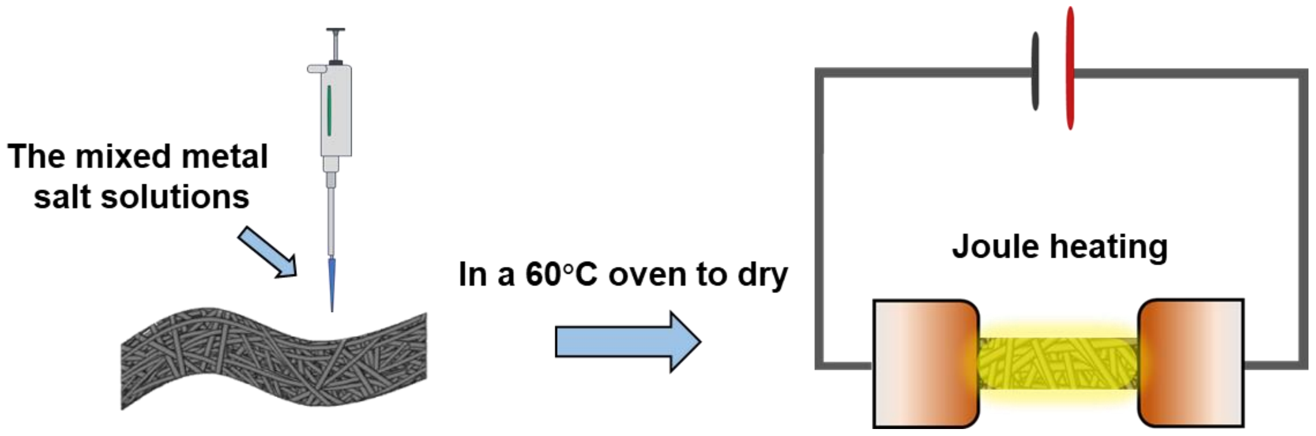

Figure S1. Synthesis scheme of HEAs and other catalysts.

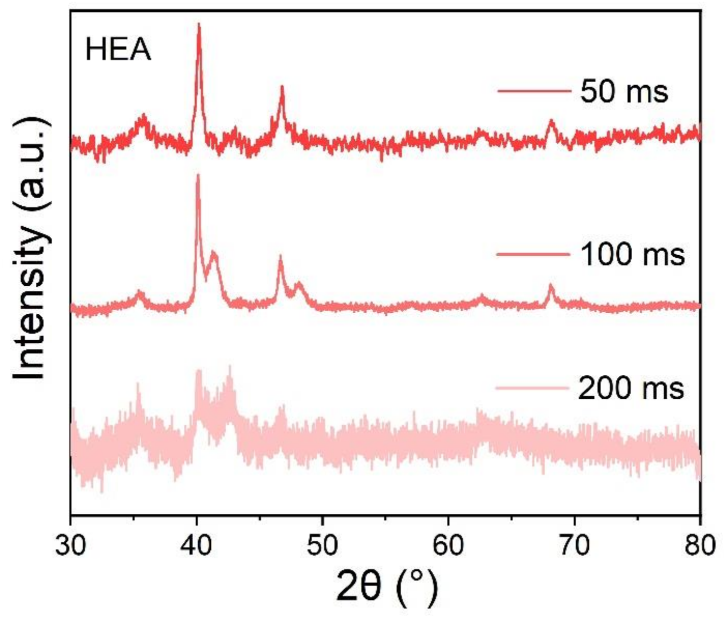

Figure S2. XRD patterns of HEA synthesized at 50 ms, 100 ms and 200 ms.

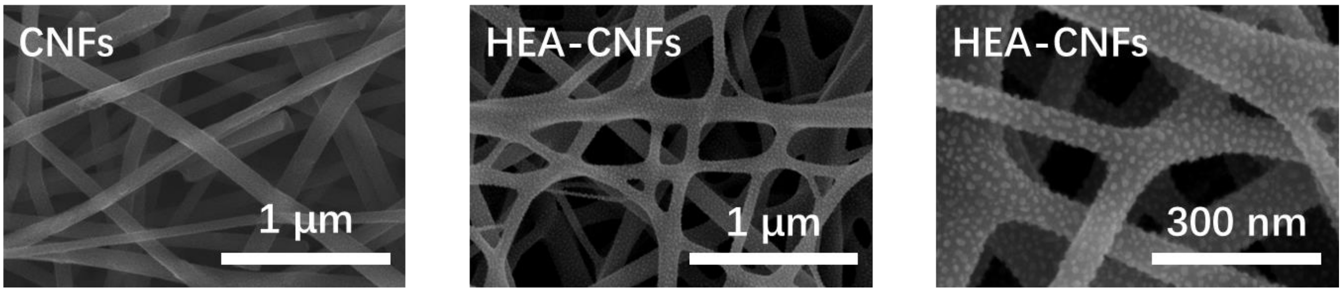

Figure S3. Scanning electron microscope (SEM) images of CNFs and the HEA nanoparticles loaded on CNFs.

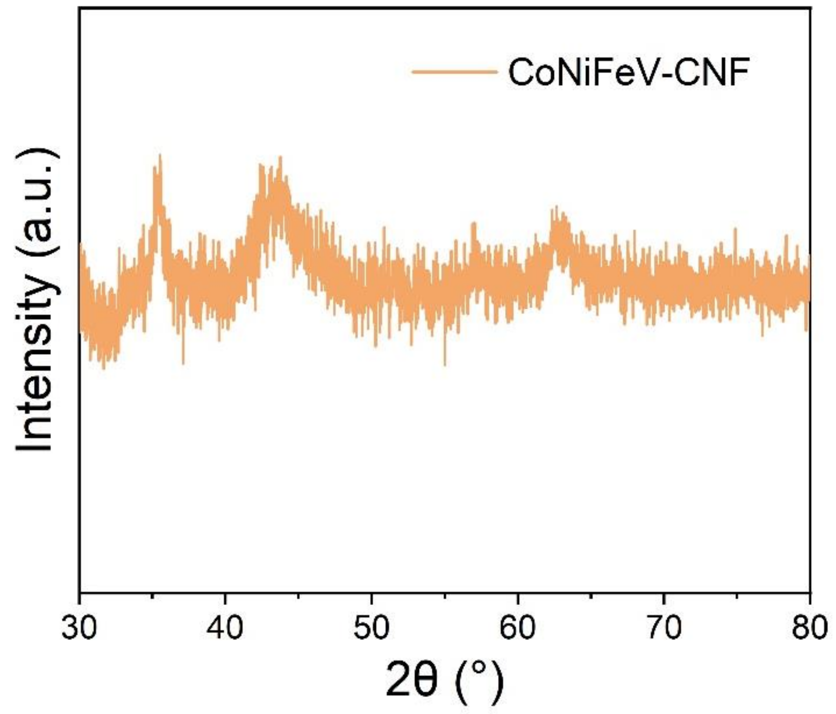

Figure S4. XRD patterns of CoNiFeV-CNF.

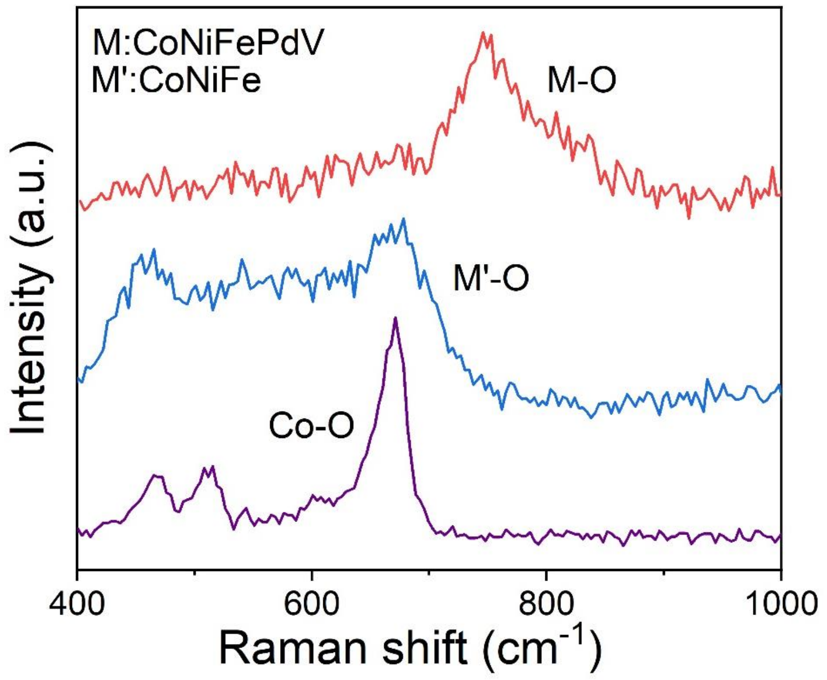

Figure S5. Raman spectra of HEA−CNF, CoNiFe−CNF, Co−CNF.

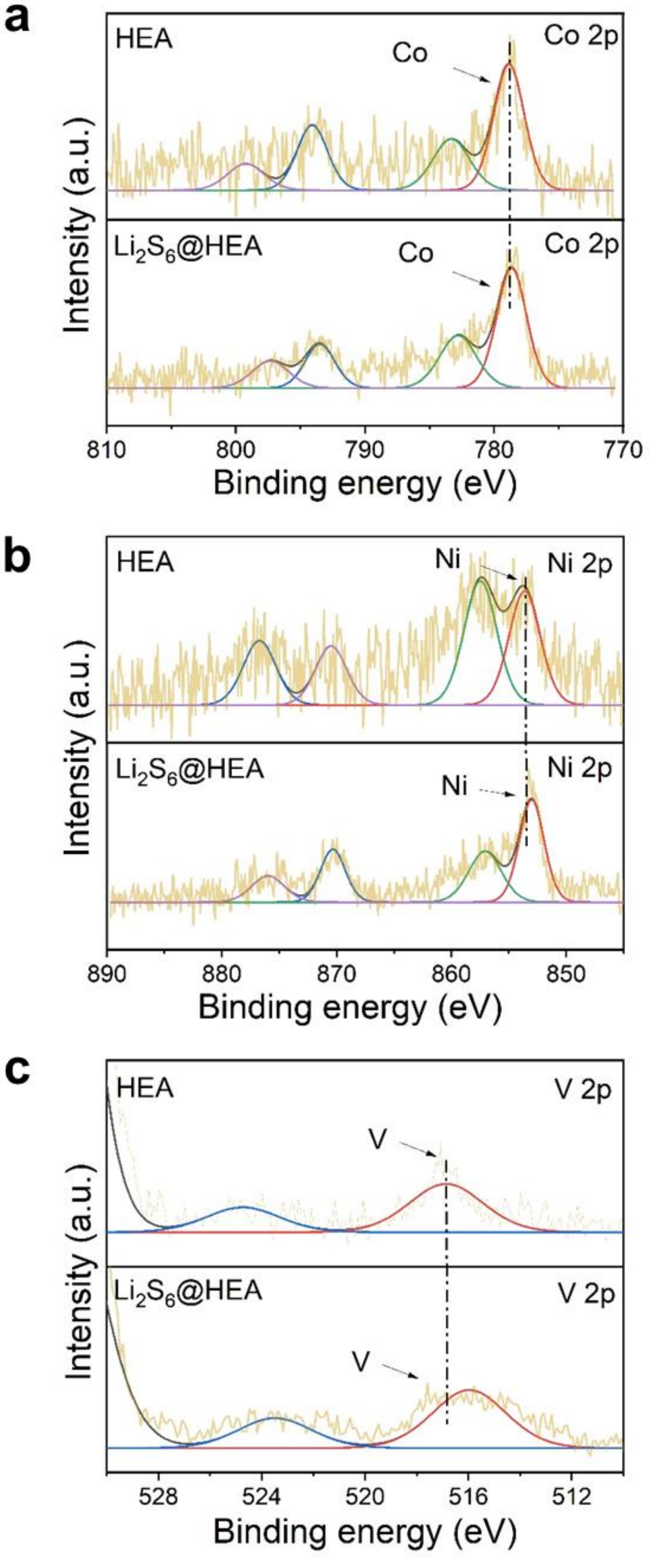

Figure S6. XPS spectra of a) Co 2p, b) Ni 2p, c) V 2p of HEAs before and after adsorption tests.

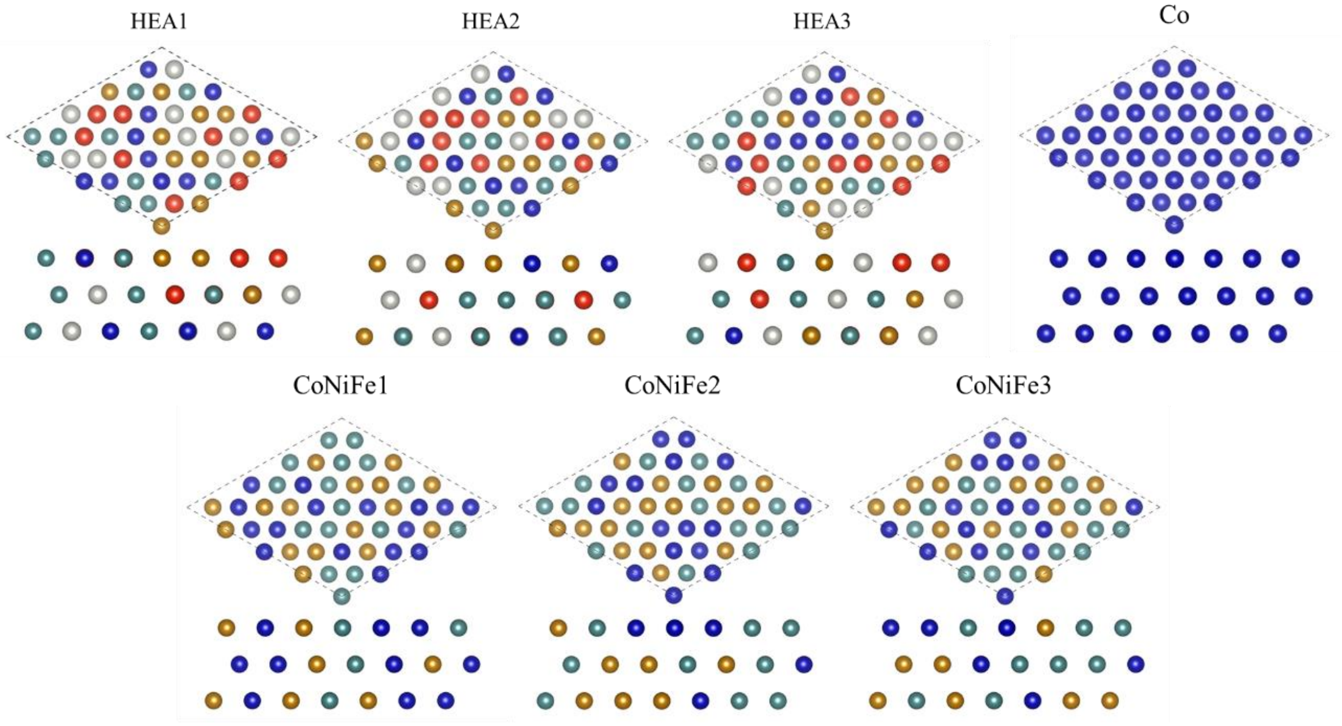

Figure S7. Initial configurations of HEA, Co and CoNiFe.

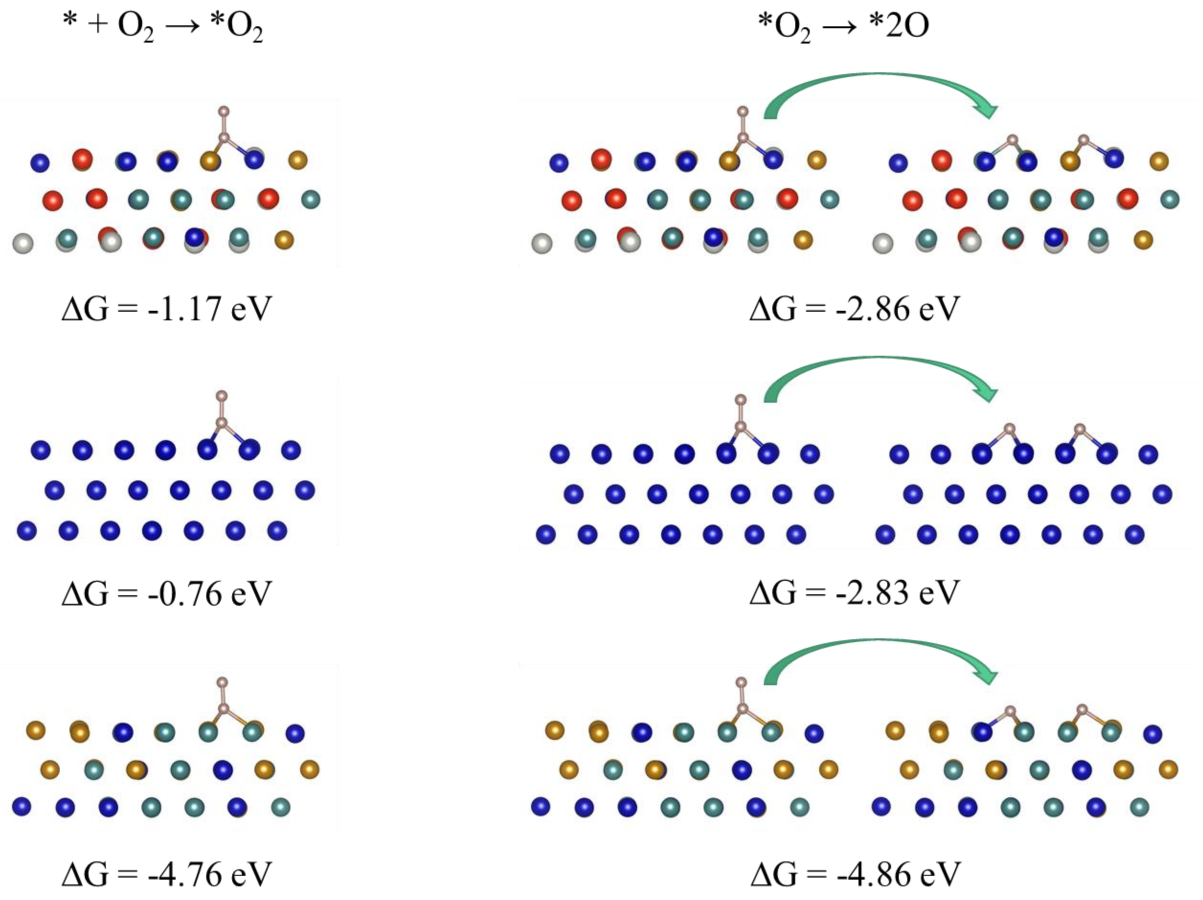

Figure S8. The reaction process of $*O_2 \rightarrow *2O$ and O_2 adsorption on HEA, Co and CoNiFe.

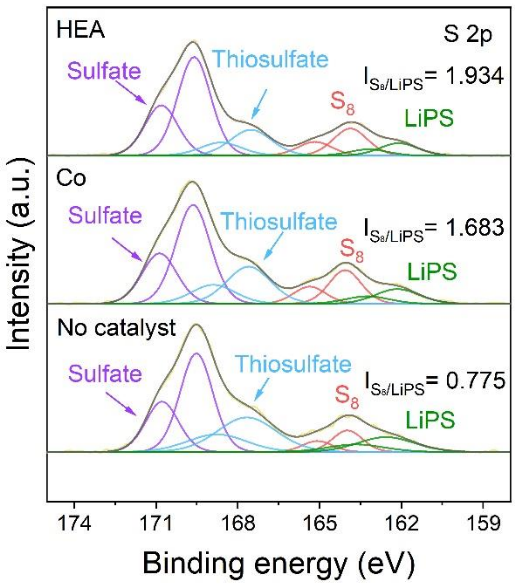

Figure S9. S_{2p} XPS spectra of HEA-based cathode, Co-based cathode and CNT/S cathode for the 1st cycle at 0.2 C.

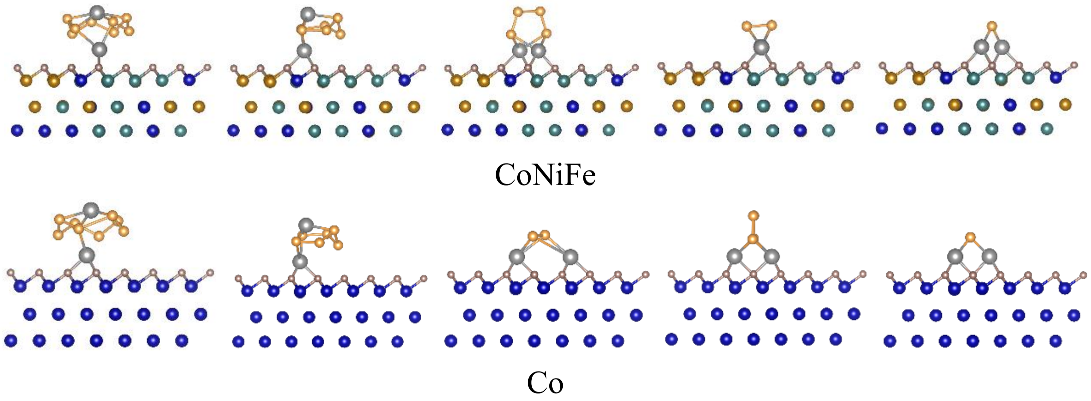

Figure S10. Optimized adsorption geometries of sulfur species adsorbed on CoNiFe and Co.

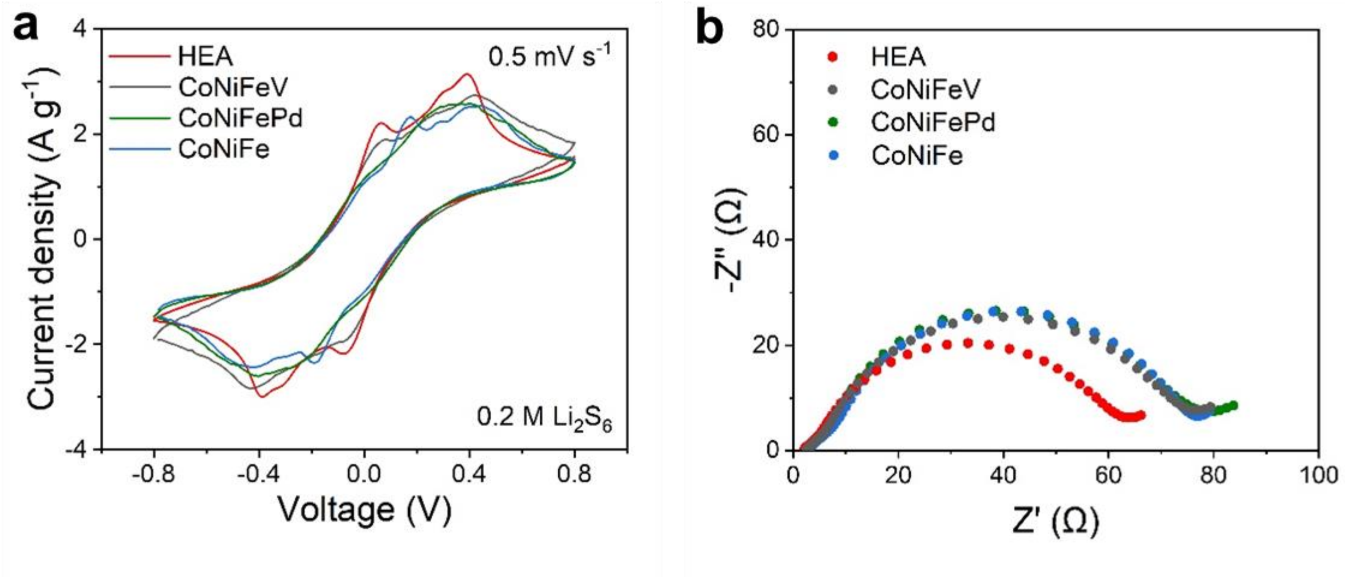

Figure S11. a) CV curves and b) EIS plots of different symmetrical cells.

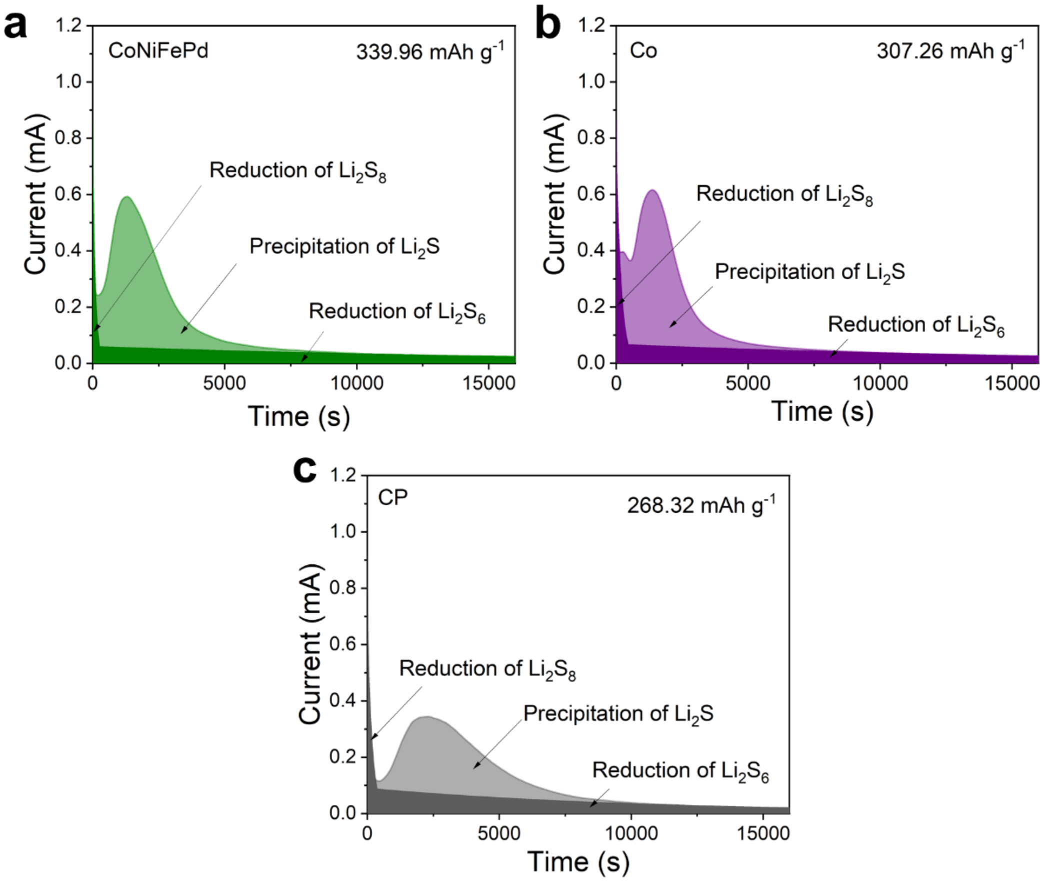

Figure S12. Li_2S precipitation profiles of a) CoNiFePd, b) Co and c) CP electrodes.

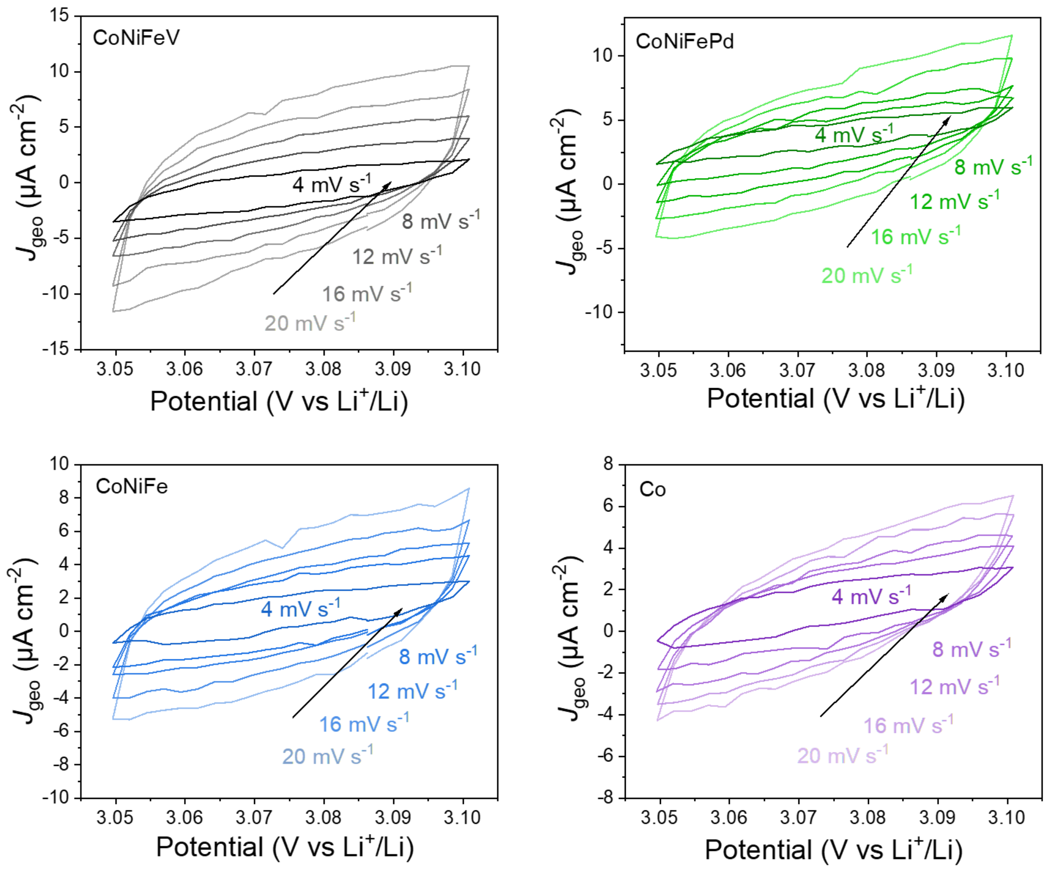

Figure S13. CV curves of CoNiFeV, CoNiFePd, CoNiFe and Co tested in the non-faradic range.

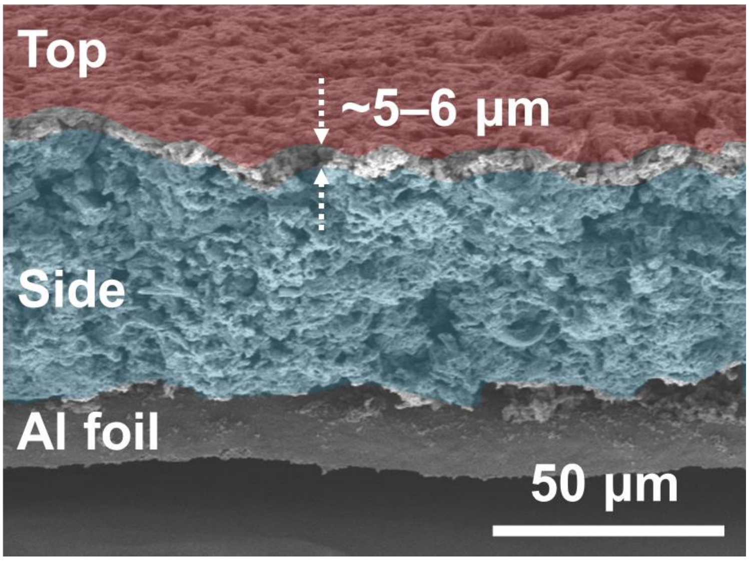

Figure S14. SEM image of S cathodes with HEAs-based interlayer.

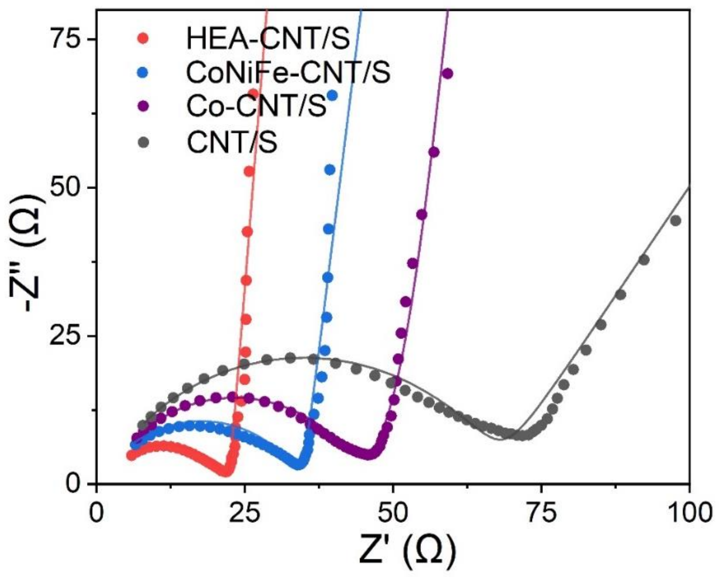

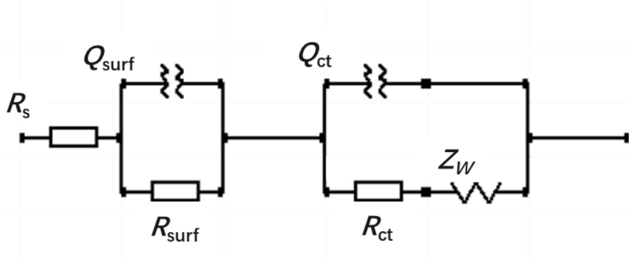

	R_s+ R_{surf}(Ω)	R_{ct} (Ω)
HEA-CNT/S	5.91	21.7
CoNiFe-CNT/S	6.6	33.91
Co-CNT/S	6.82	46.81
CNT/S	7.97	71.9

Figure S15. EIS spectra of HEA-CNT/S, CoNiFe-CNT/S, Co-CNT/S and CNT/S along with their fitting curves, corresponding equivalent circuit diagrams and the impedance of each component.

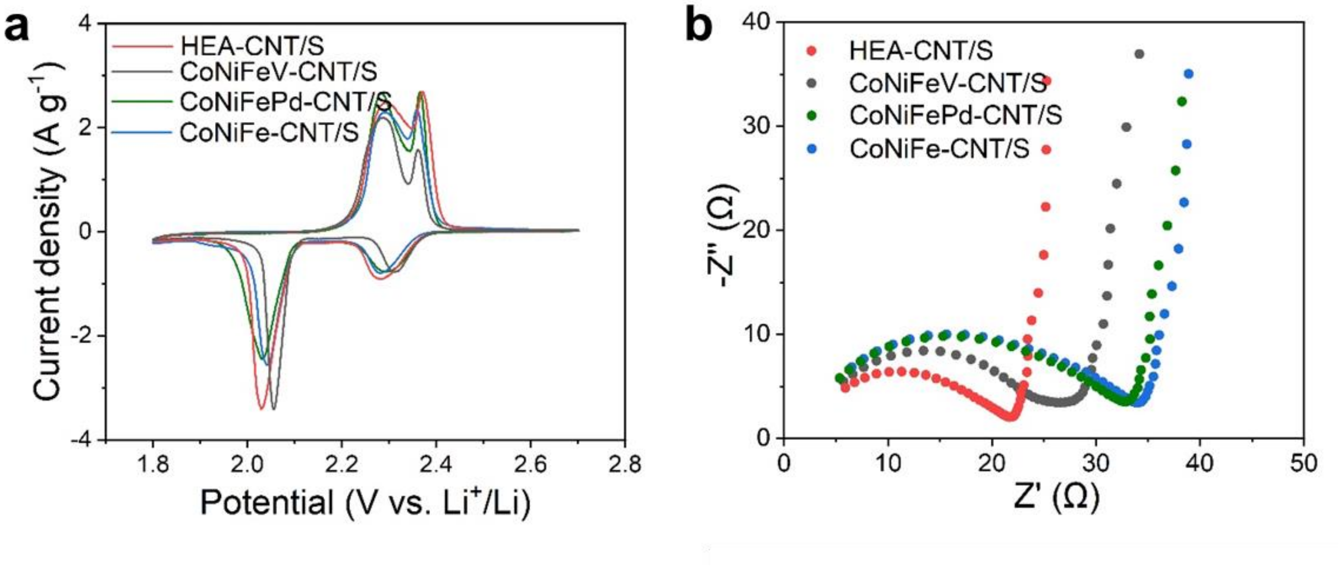

Figure S16. a) CV curves of HEA-CNT/S, CoNiFeV-CNT/S, CoNiFePd-CNT/S and CoNiFe-CNT/S at 0.1 mV s^{-1} and b) EIS spectra of HEA-CNT/S, CoNiFeV-CNT/S, CoNiFePd-CNT/S and CoNiFe-CNT/S.

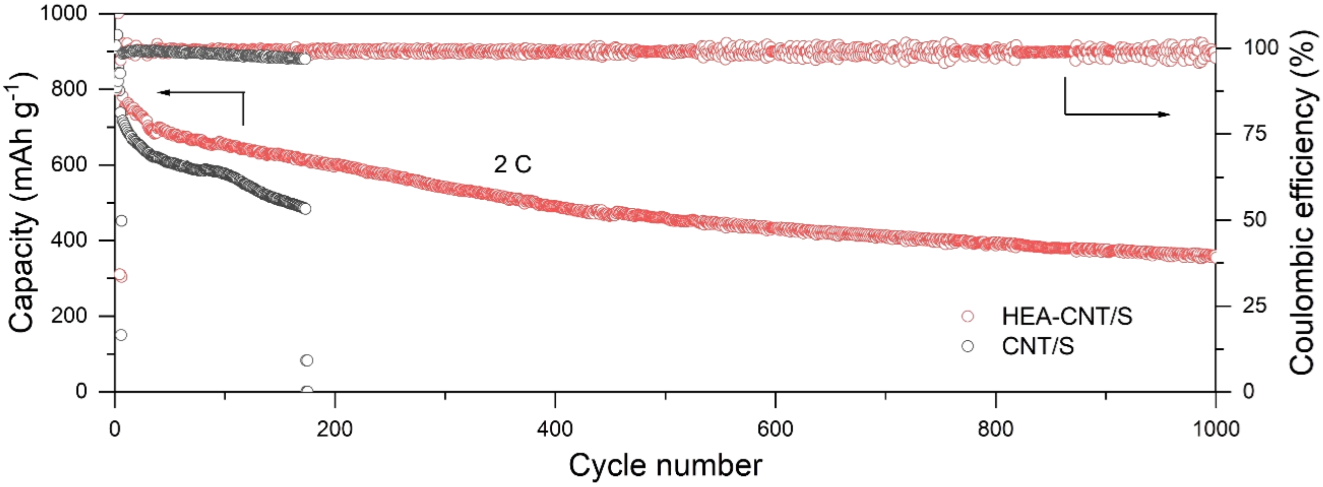

Figure S17. Pro-longed cycle life of HEA-CNT/S and CNT/S at 2 C.

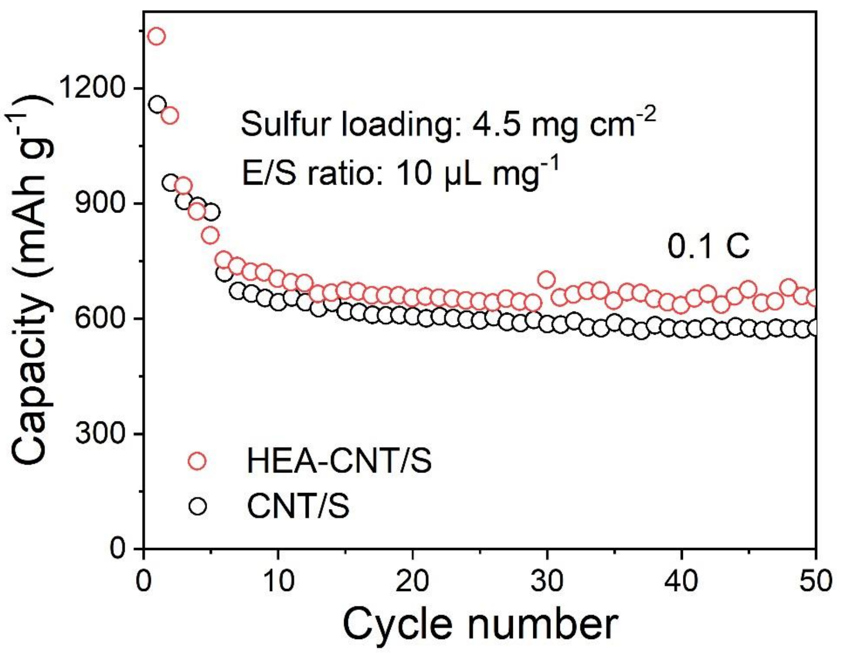

Figure S18. Cycling performance under raised sulfur loading and limited electrolyte (sulfur loading = 4.5 mg cm^{-1} and E/S=10 µL mg^{-1}).

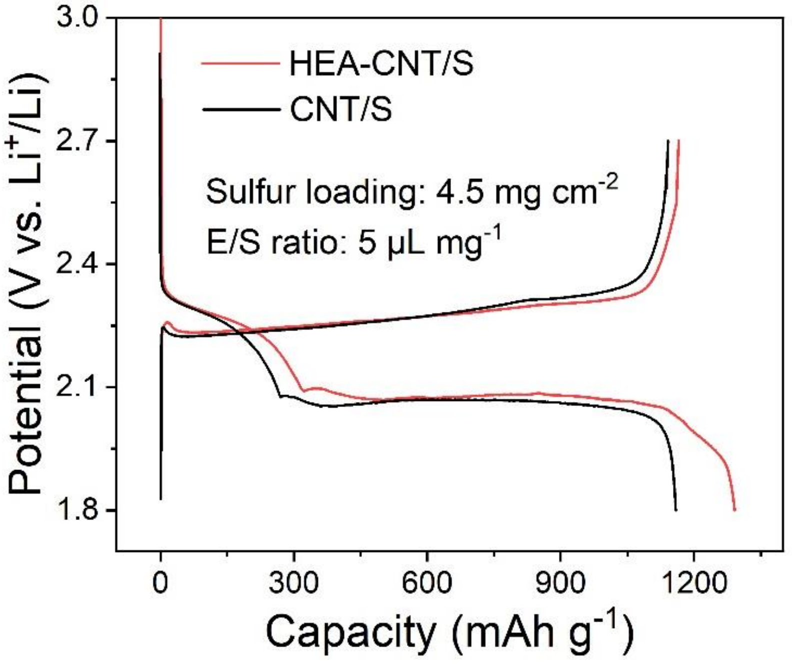

Figure S19. Charge-discharge curves of different S cathodes at 0.03 C under raised sulfur loading and limited electrolyte (sulfur loading = 4.5 mg cm^{-1} and E/S=5 μL mg^{-1}).

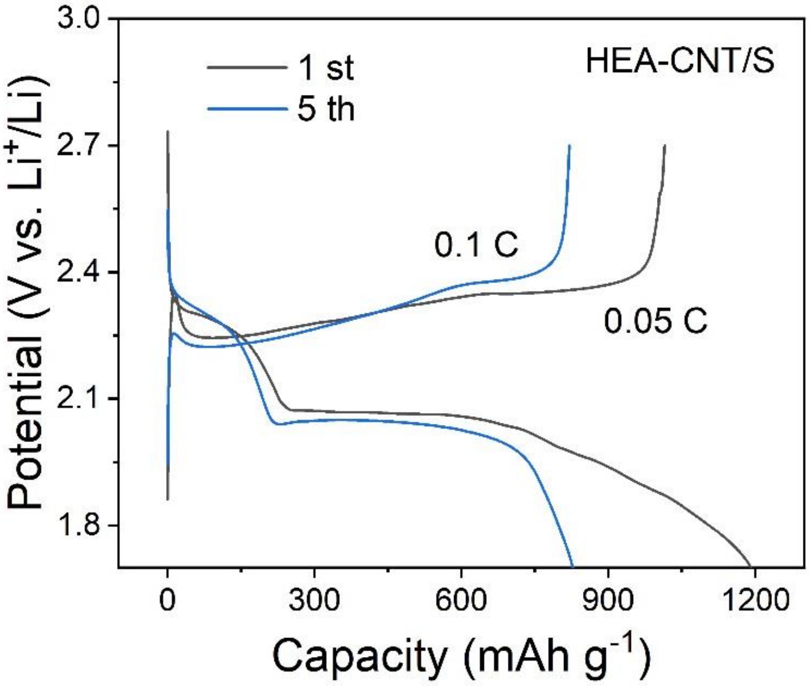

Figure S20. Charge-discharge curves of HEA-CNT/S pouch cell at 0.05 C and 0.1 C.

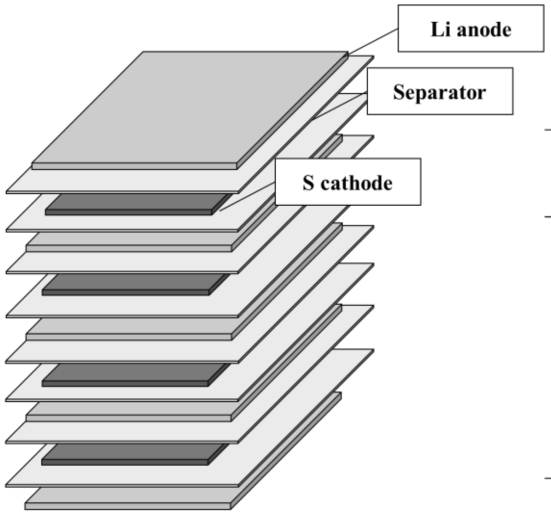

The assembly of Li-S pouch cell battery

	Li anode	Separator	S cathode	Electrolyte
	4*4 cm	4.5*4.5 cm	3.5*3.5 cm	2170 µL
	5	8	4	
Mass	425 mg	187.12 mg	775 mg	2170 mg

Figure S21. Schematic illustration of the internal structure of Li-S pouch cell and a table of the specific mass of each component.

Table S1. The elemental content in the HEA samples tested by ICP.

The element	C_x (mg kg^{-1})	Weight (%)	Atomic ratio
V	4900.5	0.49	0.9
Fe	9906.3	0.99	1.8
Co	11355.2	1.14	1.9
Ni	10555.6	1.06	1.8
Pd	11962.0	1.20	1.1

References

[1] W. Zhou, G. Wadley, A. Johnson, J. Larson, N. Tabat, A. Cerezo, K. Petford-long, W. Smith, H. Clifton, L. Martens, F. Kelly, *Acta. Mater.* **2001**, 49, 4005.

[2] N. Metropolis, A. Rosenbluth, N. Rosenbluth, A. Teller, E. Teller, *J. Chem. Phys.* **1953**, 21, 1087.

[3] G. Kresse, Furthmüller, *J. Set. Phys. Rev. B* **1996**, 54, 11169.

[4] J. Perdew, J, Chevary, S. Vosko, K. Jackson, M. Pederson, D. Singh, C. Fiolhais, *Phys. Rev. B* **1992**, 46, 6671-6687.

[5] S. Grimme, J. Antony, S. Ehrlich, H. Krieg, *J. Chem. Phys.* **2010**, 132, 154104.

[6] V. Wang, N. Xu, J.C. Liu, G. Tang, W. T. Geng, *Comput. Phys. Commun.* **2021**, 267, 108033.
